# Special Sensory Function Deficit among Patients with Post-COVID-19 Visiting a Tertiary Care Centre

**DOI:** 10.31729/jnma.8321

**Published:** 2023-11-30

**Authors:** Tika Ram Adhikari, Sonam Jamtsho, Karma Tenzin, Pelden Wangchuk, Phub Tshering, Sonam Jamtsho, Sontosh Mukhia, Dorji Penjor

**Affiliations:** 1 Department of Otorhinolaryngology, Jigme Dorji Wangchuk National Referral Hospital, Thimphu, Bhutan; 2 Audiology Unit, Department of Otorhinolaryngology, Jigme Dorji Wangchuk National Referral Hospital, Thimphu, Bhutan

**Keywords:** *anosmia*, *COVID-19*, *hearing loss*

## Abstract

**Introduction:**

Several patients who recover from COVID-19 infection continue to have persistent symptoms even after recovery from the disease. The special sensory functions such as taste, smell and hearing are affected by COVID-19 infection even after recovery from the illness. The aim of the study was to find out the prevalence of special sensory deficits among patients with post-COVID-19 visiting a tertiary care centre.

**Methods:**

A descriptive cross-sectional study was conducted among adult patients who recovered from COVID-19 visiting a tertiary care centre from 1 January 2022 to 31 December 2022. After obtaining ethical approval from the Research Ethics Board of Health, data on patients who were diagnosed with COVID-19 one year ago was obtained from the surveillance register from the Ministry of Health. They were contacted by phone call and invited to the centre to participate in the study. Appropriate clinical examination and tests were carried out to assess the special sensory deficits. A convenience sampling technique was used. The point estimate was calculated at a 95% Confidence Interval.

**Results:**

Among 271 patients, the prevalence of sensory function deficit was 39 (14.39%) (10.21-18.57, 95% Confidence Interval).

**Conclusions:**

The prevalence of special sensory deficits after recovery from COVID-19 infections was found to be similar to the findings of other studies.

## INTRODUCTION

The coronavirus disease 2019 (COVID-19) pandemic has affected people across the globe. Common symptoms among patients with COVID-19 include fever, dry cough, shortness of breath, muscle ache, confusion, headache, upper respiratory tract symptoms, gastrointestinal symptoms in addition to loss of special sensory functions like hearing, taste and smell.^1^

Loss of smell (anosmia) and alteration of taste (dysgeusia) are among the tell-tale signs of COVID-19 infections.^2^ There are several reported cases of sudden sensorineural hearing loss and vestibular symptoms following COVID-19 infection.^3-5^ Impairments of this special sensory perception have significant impacts on quality of life, and social dissociation and even contribute to unexpected potential safety issues. Some of these impairments of special sensory functions such as taste, smell, and hearing persist even after recovery from the infection.

The aim of the study was to find out the prevalence of special sensory deficits among patients with post-COVID-19 visiting a tertiary care centre.

## METHODS

A descriptive cross-sectional study was conducted in a tertiary care centre among adult patients who have recovered from COVID-19 infection from 1 January 2022 to 31 December 2022 and visited Jigme Dorji Wangchuk National Referral Hospital, Thimphu, Bhutan. Data from the Ministry of Health surveillance register was collected to trace the patients residing in the locality who were diagnosed with COVID-19 at least 1 year ago and recovered from the infection. Ethical approval for this study was granted by the Research Ethics Board of Health (Reference number: 2021/126) and administrative approval was sought from the Ministry of Health. Informed consent was obtained from the patients. The COVID-19-recovered patients were contacted via phone to participate in the study. All those who were over 18 years old and gave informed written consent were recruited into the study. Patients with active COVID-19 infections, degenerative neurological disease and a previous history of anosmia, dysgeusia, or hard of hearing were excluded from the study. A convenience sampling method was used. The sample size was calculated using the following formula:


$$\begin{array}{ll} \text{n} & ={\text{Z}}^{2}\times \frac{\text{p}\times \text{q}}{{\text{e}}^{\text{2}}}\\ & =1.96^{2}\times \frac{0.50\times 0.50}{0.07^{2}}\\ & =196 \end{array}$$

Where,

n = minimum required sample size
Z = 1.96 at 95% of Confidence Interval (CI)
p = prevalence taken as 50% for maximum sample size calculation
q = 1-p
e = margin of error, 7%

The minimum sample size was calculated to be 196. Adding a 20% non-response rate, the calculated sample size becomes 236. However, 271 patients were taken.

As per the Ministry of Health data, about 2,500 patients have recovered from COVID-19 infections in Bhutan, of which about 500 were in Thimphu. All those who consented to come to our institute, during the study period were invited to participate in the study. A total of 383 patients came for the study. After rigorous screening with inclusion and exclusion criteria, a total of 271 COVID-19-recovered patients were recruited in the final study. Appropriate clinical examination and tests were carried out. Appropriate treatment was provided as indicated.

The risk factors for loss of smell and taste were assessed with pre-designed proforma to make sure that the persistent symptoms of long COVID were not due to pre-existing conditions. The self-reported problems with taste, smell, and hearing loss before the onset of COVID-19 illness (pre-COVID) and after the recovery from COVID-19 (post-COVID) were assessed.^6^ A numeric scale of 1-10 was used to assess the smell and taste and compared the pre-COVID score to the current score (0 no sense of smell, 10 normal sense of smell/taste).^7^ Questionnaire of Olfactory DisordersNegative Statements (QOD-NS) was used to assess the effect of olfactory dysfunction on quality of life. The hearing assessment was done using Pure Tone Audiometry (PTA), impedance audiometry, and an otoacoustic emissions (OAE) test. A clinical examination was carried out to assess any sequelae/complications of COVID-19 infections. Questionnaires and clinical assessments were performed by different people to reduce bias in the study.

Data were entered and analysed using Microsoft Excel 2016. The point estimate was calculated at a 95% CI.

## RESULTS

Among 271 post-COVID-19 patients, the prevalence of special sensory function deficit was 39 (14.39%) (10.21-18.57, 95% CI).

**Table 1:** Persistent loss of special sensation after 1 year of COVID-19 infection (n = 39).

Special sense	n (%)
Anosmia/hyposmia	28 (10.33)
Dysgeusia/ageusia	2 (0.74)
Hearing loss	9 (3.32)

Among the 30 patients who had a persistent loss of taste or smell sensation, the sQOD-NS score was used to assess the effect of olfactory dysfunction on quality of life. It was found that quality of life is impacted by loss/altered sense of smell and taste sensation (Table 2).

**Table 2:** Quality of life among patients who had a persistent/altered sense of smell or taste (n= 30).

Severity	Isolate socially n (%)	Negative daily activity n (%)	More irritable n (%)	Eat out less n (%)	Loss of appetite n (%)	Effort to relax n (%)	Not normal n (%)
Strongly agree	-	2 (6.67)	2 (6.67)	-	-	-	-
Agree	1 (3.33)	3 (10)	4 (13.33)	8 (26.67)	11 (36.67)	6 (20)	3 (10)
Do not agree	1 (3.33)	3 (10)	2 (6.67)	6 (20)	2 (6.67)	7 (23.33)	4 (13.33)
Disagree	28 (93.33)	22 (73.33)	22 (73.33)	16 (53.33)	17 (56.57)	17 (56.57)	23 (76.67)

## DISCUSSION

Among 271 patients, the prevalence of persistent sensory function deficit was found in 39 (14.39%). Among the persistent sensory function deficit, 28 (10.33%) patients had a persistent loss of smell and 2 (0.74%) had a persistent loss of taste. Our study found that 30 (11.07%) participants who had a persistent loss of smell or taste had a poor quality of life. As expected, loss of appetite 11 (36%), followed by eating out less 8 (26.6%) were among the most experienced encounters that were agreed upon and rated. This could be the underlying factor for judging life as more irritable 4 (20%).

COVID-associated anosmia is due to a peripheral cause and it recovers gradually over a period of time. A study found that recovery from olfactory dysfunction depends on the severity of the COVID-19 infection, and the anosmia recovered with time.^8^ Another study conducted a long-term follow-up and concluded that after 1 year, anosmia recovered by 10% compared to 6 months recovery rate.^9^ Studying the long-term implication of COVID-19 on smell is important as it helps in counselling and smell training which helps in the recovery of smell.

In our study, we found that 9 (3.32%) patients had persistent hearing loss. Although quantitively insignificant, sensorineural hearing loss remains irreversible in most conditions and affects quality of life throughout, it requires timely intervention. Therefore, screening for hearing loss following COVID-19 is suggested to ensure effective management during the treatment window thereby addressing hearing loss-associated morbidity.^3^ Despite the abundance of literature on COVID-19 and the various associated symptoms, discussions on the relationship between COVID-19 with hearing loss and tinnitus incidence although common are rarely highlighted. Unlike earlier types of coronavirus (SARS and MERS), a rapid systematic review found that SARS-CoV-2 has been reported with records of audio-vestibular symptoms although quantitatively insignificant.^5^ A case of significant unilateral sensorineural hearing loss confirmed post-COVID on audiogram was treated with a course of oral steroids, but unfortunately with very insignificant improvement.^10^ COVID-19 may have otologic manifestations including sudden SSNHL, aural fullness, vertigo, and intra-labyrinthine haemorrhage. There is new evidence of COVID-19 demonstrating a reduction of the amplitude of the emissions in transient-evoked otoacoustic emissions (TEOAE) signifying disturbances in cochlear hair cells.^4^

In our study 2 (0.74%) patients had persistent dysgeusia. Ageusia (loss of taste sensation) or dysgeusia (altered sense of smell) is one of the characteristic symptoms of COVID 19-infection. It could be due to loss of sense of smell. However, it is also postulated that the angiotensin-converting enzyme 2 receptor, which is the main host cell receptor of SARS-CoV-2 for binding and penetrating cells, is widely expressed in epithelial cells of the oral mucosa. Damage of mucosal epithelial cells of the oral cavity during the early stage of the disease leads to loss of taste sensation.^4^ Other possible mechanisms are hypoxia and zinc deficiency.^11^

Long COVID condition is broadly defined as signs, symptoms, and conditions that continue or develop after initial COVID-19 infection. The signs, symptoms, and conditions are present four weeks or more after the acute phase of infection, may be multisystemic and may present with a relapsing-remitting pattern and progression or worsening over time, with the possibility of severe and life-threatening events even months or years after infection. Change of smell and taste is a common long COVID symptom.^12^ This study supported the above theory of long-term COVID conditions and found that 14% of the patients who recovered from COVID-19 infection had persistent symptoms.

Among the patients who had persistent symptoms, we assessed the quality of life using (sQOD-NS) which is reliable and validated in previous studies.^13^ It was found that quality of life is significantly impacted by loss/altered sense of smell and taste sensation. It was also interesting to note that patients who did not have loss of smell or taste but had other symptoms such as nasal congestion had impacted the quality of life.

The strength of this study is it is the only study conducted in Bhutan to assess sensory function post-COVID-19 infection. This piece of literature can potentially add evidence at the global level and act collectively with the sensitization and advocation for the prevention and timely intervention concerning the long-term sequelae of COVID-19 infection.

There are several limitations to this study. Subjective assessment was used which can have recall bias. The sample size is not very big and only the people residing within the locality could participate in the study.

## CONCLUSIONS

The prevalence of loss of special sensory function after a COVID-19 infection was similar to other international studies. The quality of life is significantly impacted even after COVID-19 recovery with loss of sensory functions such as smell, taste and hearing. A study with a greater range of participants will help us better understand more about the loss of special sensory function and its management.
